# Evaluation of Erythema Severity in Dermatoscopic Images of Canine Skin: Erythema Index Assessment and Image Sampling Reliability

**DOI:** 10.3390/s21041285

**Published:** 2021-02-11

**Authors:** Blaž Cugmas, Daira Viškere, Eva Štruc, Thierry Olivry

**Affiliations:** 1 Biophotonics laboratory, Institute of Atomic Physics and Spectroscopy, University of Latvia, 19 Raiņa Blvd., LV-1586 Rīga, Latvia; daira.viskere@lu.lv; 2 Faculty of Veterinary Medicine, Latvia University of Life Sciences and Technologies, 8 Kristapa Helmaņa Str., LV-3004 Jelgava, Latvia; 3 Vetamplify SIA, veterinary services, 57/59-32 Krišjāņa Valdemāra Str., LV-1010 Rīga, Latvia; eva@vetamplify.com; 4 Department of Clinical Sciences, College of Veterinary Medicine, NC State University, 1060 William Moore Dr., Raleigh, NC 27607, USA; tolivry@ncsu.edu; 5 Comparative Medicine Institute, NC State University, Raleigh, NC 27606, USA

**Keywords:** canine atopic dermatitis, smartphone dermatoscopy, erythema severity, erythema index, dogs, disease severity scales, CADESI4, intraclass correlation coefficient, multispectral imaging, image sampling

## Abstract

The regular monitoring of erythema, one of the most important skin lesions in atopic (allergic) dogs, is essential for successful anti-allergic therapy. The smartphone-based dermatoscopy enables a convenient way to acquire quality images of erythematous skin. However, the image sampling to evaluate erythema severity is still done manually, introducing result variability. In this study, we investigated the correlation between the most popular erythema indices (EIs) and dermatologists’ erythema perception, and we measured intra- and inter-rater variability of the currently-used manual image-sampling methods (ISMs). We showed that the *EI_BRG_*, based on all three RGB (red, green, and blue) channels, performed the best with an average Spearman coefficient of 0.75 and a typical absolute disagreement of less than 14% with the erythema assessed by clinicians. On the other hand, two image-sampling methods, based on either selecting specific pixels or small skin areas, performed similarly well. They achieved high intra- and inter-rater reliability with the intraclass correlation coefficient (ICC) and Krippendorff’s alpha well above 0.90. These results indicated that smartphone-based dermatoscopy could be a convenient and precise way to evaluate skin erythema severity. However, better outlined, or even automated ISMs, are likely to improve the intra- and inter-rater reliability in severe erythematous cases.

## 1. Introduction

**Figure 1:**
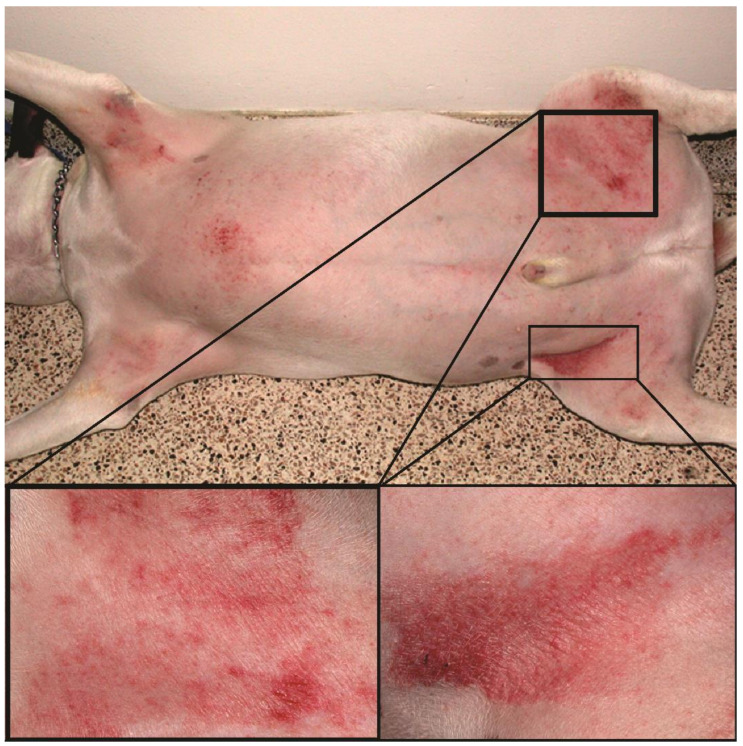
Atopic dog’s skin in the inguinal region with severe erythema and excoriations.

Canine atopic dermatitis (AD) is a chronic allergic and inflammatory skin disease with characteristic clinical features [1]. It is one of the most common skin diseases in dogs, with a prevalence of 3–15% [2]. Environmental and food allergens trigger the allergic reaction, manifesting as pruritus (i.e., itch) and skin lesions that include erythema (redness), hyperpigmentation (increased pigmentation), and excoriations (scratched lesions) (Figure 1). In most dogs, AD is a lifelong condition that requires long-term management, including the administration of antipruritic and anti-inflammatory drugs, allergen immunotherapy, and good hygiene of the coat and skin [3]. Since the treatment response is highly individual, the precise tracking of the evolution of clinical signs is crucial to select the proper anti-allergic therapy.

Few disease severity scales have been developed to grade the clinical signs of canine AD. First, an owner-assessed pruritus estimation is done with a 10-point Pruritus Visual Analog Scale (PVAS) [4]. Skin lesions are evaluated most often with the fourth iteration of the Canine Atopic Dermatitis Extent and Severity Index (CADESI4) [1]. The CADESI4 is based on the grading of different lesion types on 20 locations leading to 60 assessments across several AD-related body sites; its execution takes approximately 4 min. Since the CADESI4 is not very sensitive to short-term changes of chronic skin lesions, such as hair loss or increased skin thickness, the scale’s derivative-based only on erythema evaluation has been proposed [5].

Still, erythema and other skin lesions are evaluated visually, making a final score firmly subjective. Factors including illumination (light temperature, brightness, and vignetting), native skin color, and medical experience can affect the final score [6]. For example, CADESI4 scores were only moderately correlated (Spearman’s *r_s_* = 0.48) between the inexperienced raters [1]. A slightly better reliability was achieved by the use of a different graphic scale, the 2D investigator’s global assessment (2D-IGA) instrument (*r_s_* = 0.96, Cohen’s kappa (*K_C_*) = 0.90 for intra- and *r_s_* = 0.75, *K_C_* = 0.53 for inter-rater reliability, respectively) [7]. In humans, there are studies on intra- and inter-rater reliability when erythema was the only lesion scored (in person or optically). For the intra-rater reliability, the following parameters were reported: intraclass correlation coefficients (ICC) = 0.55–1.00 [8,9,10,11] and Fleiss’ kappa (*K_F_*) = 0.69 [8]. The inter-rater reliability was, as expected, lower, with the reported ICC = 0.41–0.78 [8,12], *K_C_* = 0.18–0.51 [13], and *K_F_* = 0.71 [8].

Various custom-made or commercial imaging or spectroscopic devices (e.g., Mexameter MX 18, Courage-Khazaka Electronic, Köln, Germany) tried to overcome the subjectivity of erythema evaluation [6,14,15,16,17] by calculating an erythema index (EI), which is a ratio between those spectral or imaging components that correlate well with the skin redness. A quotient between the red and green colors is the most common since the skin pigment melanin has a smaller impact on these two channels. On the contrary, some authors demonstrated the benefits of an added blue channel [18,19,20]. In general, most studies [6,19,21,22,23,24] presented a high correlation between the visual and optical erythema estimations with the correlation coefficients from 0.69 to 0.91. Furthermore, there was a good to excellent agreement between various optical devices with the Pearson’s correlation coefficient (*r_p_*) of 0.76–0.81 [14] and the coefficient of determination (*r^2^*) between 0.82 and 0.99 [25,26]. On the other hand, two facial devices for skin analysis (VISIA, Canfield, Parsippany, NJ, USA and CSKIN, source unknown) achieved a poor correlation with visual scores or between themselves (*r_p_* = 0.21–0.49) [27].

Discrepancies among devices probably appear due to manual skin sampling. Many spectroscopic systems with single-point probes average the erythema intensity in a small area ranging between 0.2 and 0.5 cm^2^ for commercial devices like the Mexameter M X18 (Courage-Khazaka Electronic, Köln, Germany), DermaSpectrometer (Cortex Technology, Hadsund, Denmark), and Chromameter CR 200 (Minolta, Osaka, Japan) [14]. Most imaging systems have a large sampling area (e.g., 3.0 and 7.1 cm^2^ for the dermatoscope DermLite DL1 (3Gen, San Juan Capistrano, CA, USA), or the custom-made Skimager [19,21]), which can produce faulty EI readings due to the inclusion of hair and pigment. Therefore, only a suitable skin area needs to be selected from the acquired images. Currently, specialists sample skin images manually by selecting specific pixels or small areas [17,19,21]. On the other hand, there are a few semi-automated approaches [6,28], where the user would select erythematous and native skin areas with the representative erythematous skin being determined by the redness gradient- or fuzzy entropy-based algorithm.

As of today, erythema indices (EIs) and manual image-sampling methods (ISMs), which can significantly impact erythema estimation, have not been studied and compared thoroughly. With this study, we wanted to demonstrate that a smartphone-based dermatoscopy, relying on certain EIs and ISMs, could be a convenient and reliable method for evaluation of the skin erythema severity in dogs with AD. Therefore, we first investigated a correlation between the most common EIs, including the a* dimension of the CIELAB color space, and visual erythema scores. Secondly, we applied three different ISMs on erythematous skin images, which served to estimate intra- and inter-rater variability of the proposed optical system.

## 2. Materials and Methods

The Latvian Food and Veterinary Service approved this study under the reference number 1.1-13E/20/865. We enrolled 43 purebred or crossbred client-owned dogs, which were presented at the dermatology service with AD diagnosis during a three-week period. The average age was 6.8 years (0.4–18.5 years). The most common breeds were American Staffordshire terrier (*n* = 5), Shih Tzu, Boston terrier (3), Labrador retriever, pointer, West Highland white terrier, and English and French bulldogs (2). We evaluated erythema in the inguinal region, which had to exhibit a low amount of hair and no secondary lesions (e.g., lichenification, excoriation, and hyperpigmentation). On the day of measurement, we made sure that the measurement site had not been washed or treated (e.g., with lotions, shampoos, etc.).

**Figure 2:**
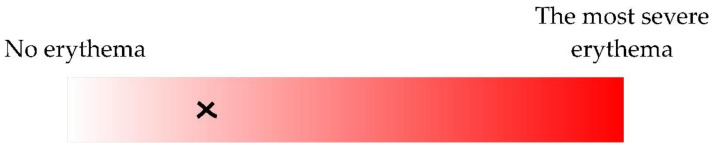
The severity of erythema was estimated according to the continuous scale (manual valuation was transformed to a percentage; 0–100%). The example of an erythema estimation (25.07%) is shown as a cross.

**Figure 3:**
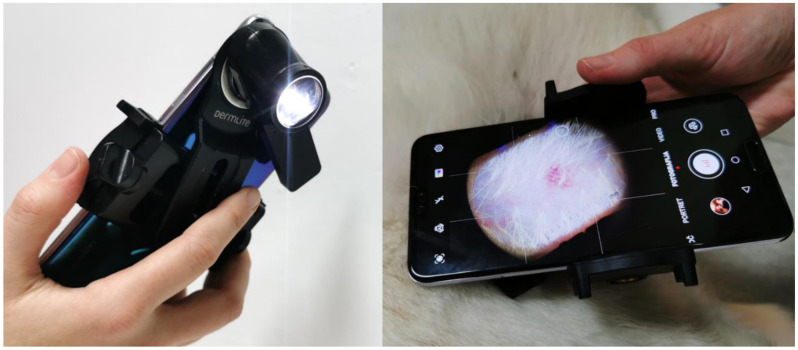
Optical erythema evaluation relied on the smartphone-based dermatoscopic system.

Two different erythema evaluations were made:

(1) Visual; according to the continuous erythema scale (Figure 2). Three different dermatology residents were involved in marking a spot corresponding to the severity of skin erythema. However, only the one being the patient’s clinician performed the assessment.

(2) Optical (Figure 3); from the images acquired by an optical system with a smartphone (Nokia 6, v.2017, HMD Global, Espoo, Finland) and a dermatoscope (DermLite DL1 basic, 3Gen Inc.). The exposure time was locked on the patch 20 (neutral 8 (.23*), L* = 81.3) of the ColorChecker Classic (X-rite, Grand Rapids, MI, USA). Jpeg images (90% quality, 4608 × 3456 pixels) were acquired in the program Open Camera (v1.47.3, Mark Harman, Cambridge, UK) with the following settings: photo mode (STD), white balance (manual, daylight), scene mode (steady photo), color effect (none), ISO (50), and focus (auto). The camera was held 3.1 cm away from the skin, resulting in a circular 3.0 cm^2^ sampling area. One operator performed all the acquisitions independently from the visual assessment.

**Figure 4:**
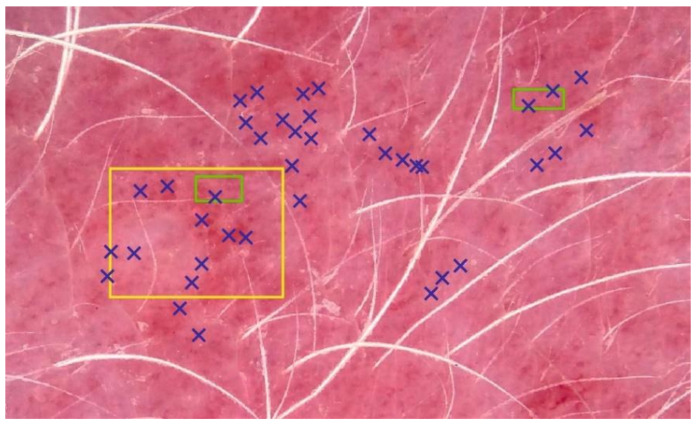
Three image-sampling methods (ISMs). Erythema index (EI) calculation was based on 60 pixels (PT, blue), and all the pixels in two small (SQ2, green) or one big square (SQ1, yellow). For the sake of simplicity, only a few selected pixels are shown for the PT method.

After the acquisition, RGB (red, green, blue color space) images were normalized against the white standard. Three new raters (veterinarians, different from the dermatologists in the visual erythema evaluations), performed three manual image-sampling methods (ISMs) in order to select a representative portion of erythematous skin without depigmented or pigmented spots and hair (Figure 4). Each of raters executed image sampling twice. At least one month passed between the original and a repeated image sampling. The first ISM included manually selecting 60 representative pixels (PT). Secondly, pixels from two small (SQ2) or one large square (SQ1) were considered. Typical blue (*B*), green (*G*), and red (*R*) values were calculated as an average from all the selected pixels. Different EIs were estimated as:

(1) $${\mathrm{EI}}_{\mathrm{RG}}=\frac{R}{G}$$

(2) $${\mathrm{EI}}_{\mathrm{GR}}=\frac{G-R}{G+R}$$

(3) $${\mathrm{EI}}_{\mathrm{BRG}}=\frac{\sqrt{B\cdot R}}{G}$$

(4) $${\mathrm{EI}}_{\mathrm{BG}}=\frac{B}{G^{2}}$$

The pixels’ RGB values were additionally used for a calculation of the dimension a* of the CIELAB color space according to the following model:

(5) $$a^{*}=a_{0}+a_{1}R+a_{2}G+a_{3}B,$$

where *a*_0–3_ are regression coefficients, retrieved from the calibration procedure on all patches of the ColorChecker Classic [29].

First, and separately for each EI and ISM, we evaluated the relationship between visual and optical erythema estimation by calculating Spearman’s rank correlation coefficient (*r_s_*) and residuals of linear regression. Based on the six ISM executions, we estimated the mean and standard deviations, which served to select the best-performing EI and ISM. Finally, we studied intra- and inter-rater reliability among all three raters (veterinarians) by calculating Spearman’s rank correlation coefficient (*r_s_*), intra-class correlation *ICC (2, k)* (two-way random effects, absolute agreement, multiple raters [30]), and Krippendorff’s alpha (*α_k_*). Additionally, we studied absolute intra- and inter-rater agreements for *EI_BRG_* and a*.

## 3. Results

We optically and visually estimated the erythema severity in 43 dogs. We excluded two measurements from further analysis due to extreme skin thinness. For most of the EIs and ISMs, we found a strong correlation between optical and visual assessments (Table 1). As shown in our preliminary study [21], the *EI_BRG_* achieved the best performance with an average Spearman’s correlation coefficient (*r_s_*) of 0.74. Due to the decreasing negative numerator’s value in Equation (2), the *EI_GR_* exhibited a negative correlation. The single best and worse correlations between optical and visual erythema assessment resulted in *r_s_* of 0.83 (rater 1, based on *EI_BRG_* and SQ2) and 0.55 (rater 2, *EI_BG_*, SQ1), respectively. Selecting specific representative image pixels (method PT, Figure 4) turned out to be the best performing ISM with a mean *r_s_* of 0.71. The SQ2 method, which is based on two small squares, exhibited a slightly lower correlation strength, but a faster mean execution (3.7 ± 0.3 vs. 31.2 ± 5.9 s).

The *EI_BRG_* and the PT ISM also produced the smallest mean residuals (i.e., errors) in the linear regression analysis between visual and optical erythema evaluation (Table 2). The single best and worst models resulted in the mean fitting error of 10.3% (rater 1, *EI_RG_*, PT) and 14.0% (rater 2, *EI_BG_*, SQ1), respectively. For the best-aforementioned model, the residual values ranged between −29.3% and 31.3%, with a standard deviation of 13.2%.

The agreement between raters when applying ISMs was strong, since all the studied parameters (Spearman’s rank correlation-*r_s_*, ICC, and Krippendorff’s alpha-*α_k_*) were above 0.90 (Table 3). The best and worst ISM applications resulted in an *α_k_* of 0.99 (rater 2, a*, PT) and 0.91 (rater 2, *EI_BG_*, SQ1) for intra-, and 0.98 (rater 1–2, a*, PT) and 0.70 (rater 1–3, *EI_RG_*, PT) for inter-rater reliability, respectively. When investigating individual ISMs, it seems that PT and SQ2 were more reliable for a single rater (Table 4). On the other hand, both ISMs exhibited a higher inter-rater variability compared to SQ1.

**Figure 5:**
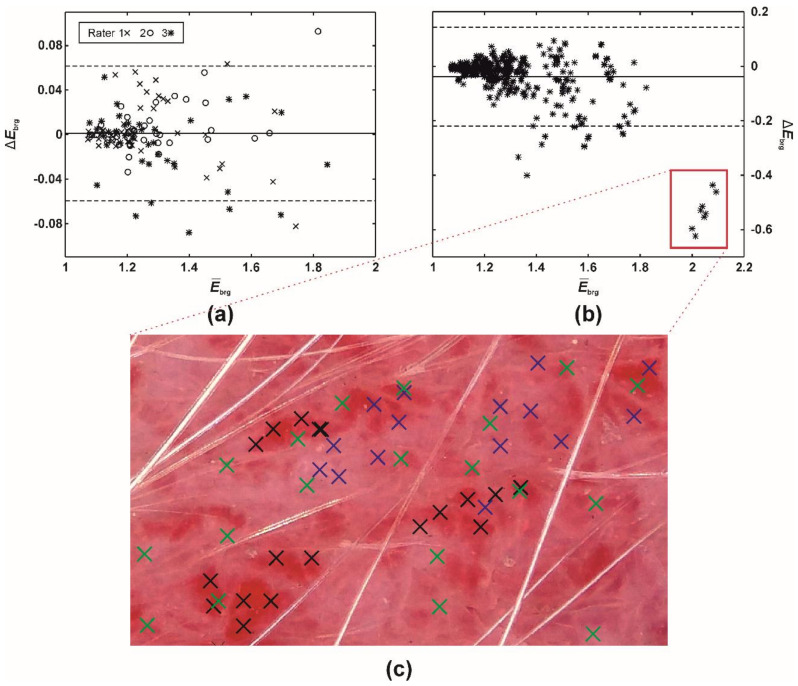
A difference (Bland-Altman) plot of *EI_BRG_* misestimates between (**a**) a single (intra-) and (**b**) multiple raters (inter-rater absolute agreement). The full and two dashed lines represent the difference mean and 95% limits of agreement (i.e., 1.96 × of SD), respectively. (**c**) Execution of PT (60 pixels), ISM. Blue (rater 1), green (rater 2), and black crosses (rater 3) mark the pixels selected by the three raters.

Despite the mean intra- and inter-rater misestimates in *EI_BRG_* being small (i.e., up to 0.05; Table 5), the further study revealed that the differences had increased along with the severity of erythema (Table 5, Figure 5). Evaluating native or mildly erythematous skin by a single rater resulted in minor *EI_BRG_* misestimates of up to 0.07 (Figure 5a). On the other hand, the maximal disagreement between multiple raters was −0.62 (Figure 5b), representing an error between ~30 and 60%. This phenomenon occurred on the skin with severe and patchy erythema (Figure 5c) where the raters selected different sampling weights for severely and mildly erythematous skin.

**Figure 6:**
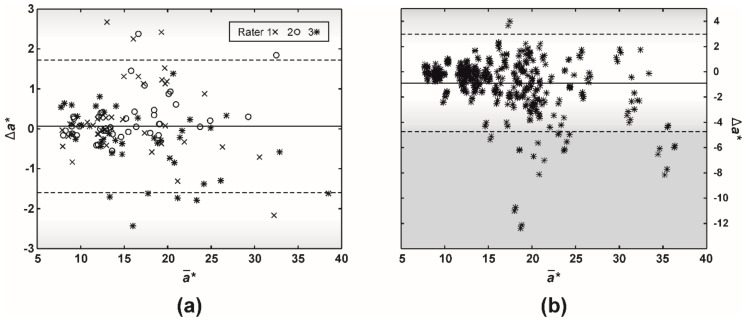
A difference (Bland-Altman) plot of a* (CIELAB color space) misestimates between (**a**) a single (intra-) and (**b**) multiple raters (inter-rater absolute agreement). The full and two dashed lines represent the difference mean and 95% limits of agreement (i.e., 1.96 × of SD), respectively. Gradients of gray color mark just-noticeable difference (JND, the range of 2.3–5.0), which corresponds to the human eye capability to spot a difference between two colors.

The study on intra-rater disagreement in a* revealed that most of the differences were below the so-called just-noticeable difference (JND, the range of 2.3–5.0), which corresponds to the human eye’s capability to spot a difference between two colors (Figure 6a). Similarly, the human eye would not have detected most of the misestimates between raters (Figure 6b). However, 4% of the ratings resulted in an a* difference larger than 5.0.

## 4. Discussion

This study, which focused on the feasibility of a smartphone-based dermatoscopy, is one of the first of its kind in veterinary dermatology. Our results confirmed the findings of our preliminary study [21], that the proposed system can present an objective and reliable method for the evaluation of the skin erythema severity in dogs with AD.

We found a strong correlation between the optical (*EI_BRG_* and PT) and the dermatologists’ visual erythema severity estimates. On average, the Spearman coefficient (*r_s_*) was 0.75 (Table 1), with a range between 0.70 and 0.83. Our results are comparable to the studies on human erythematous skin, where the obtained correlation coefficients were between 0.69 and 0.91 [6,22,24]. However, Frew et al., who reported the highest *r_p_* of 0.91, differentiated only between a few erythematous categories without the inclusion of the native skin color [6]. We should also point out that, in this study, only one dermatologist performed each visual erythema estimation without repetitions. As a result, our conclusions do not consider any possible intra- and inter-rater variability in the visual evaluation of skin erythema.

Compared to the rest of EIs, including the dimension a* of the CIELAB color space, *EI_brg_*’s performance in terms of correlation and absolute fitting residuals was superior by more than 3%. Still, some authors discourage using EIs as *EI_BRG_*, which rely on the blue channel, due to the strong blue light absorption by melanin [6]. This factor is probably the main reason why Saknite et al. [18] indicated that *EI_BRG_* is the most suitable for detecting contrast between pigmented and non-pigmented skin. Generally, pigment seems to have a significant impact on the visual perception of erythema. In a study on the inter-rater reliability of evaluating erythema visually, Zhao et al. showed that the ICC dropped from 0.41–0.78 for non-pigmented to only 0.06–0.23 for pigmented erythematous skin, respectively [12]. All these observations could discourage us from promoting *EI_BRG_*. However, the inguinal region of all 43 dogs in our study was never completely pigmented, enabling us to find and sample non-pigmented, erythematous skin.

Despite the coordinate a* corresponding with the red color in the CIELAB color space, a* did not correlate better than other EIs with the visual perception of erythema. Actually, its mean correlation coefficient and fitting residuals were lower for 0.04 and 0.6 p.p. compared to *EI_BRG_* (Table 1 and Table 2). Other studies reported even more discouraging results on the CIELAB performance. Logger et al. found a weak correlation between a visually determined erythema score and a* with *r_s_* of 0.37 [25]. Similarly, a* retrieved from RGB images exhibited a limited capability to differentiate between erythema categories in canine skin [31].

For a single rater, the ISM with two small squares (SQ2) performed the best [19,21]. However, the PT method (selecting specific pixels) achieved slightly better results when adding extra raters and ISM repetitions, but the differences were negligible (Table 1, Table 2 and Table 4). Collectively, the intra- and inter-reliabilities of ISMs were very high with the parameter values (*r_s_*, ICC, and *α_k_*) above 0.90 (Table 3). Such reliability is superior to the studies on the visual or optical evaluations of erythema in human skin, in which reliability parameters were usually well below 0.90 (see Introduction) [1,7,8,9,10,11,12,13].

Among ISMs, SQ1’s general performance was the worst. Previously, we speculated that the SQ1 method samples also hair and pigment, which would negatively affect the correlation with the visual evaluation of erythema. However, SQ1 exhibited a significantly higher inter-rater reliability with an *α_k_* of 0.93, compared to 0.88, achieved by PT and SQ2 (Table 4). As expected, with smaller skin areas or even single pixels to choose from, there is a bigger probability that a rater would sample markedly different skin with various erythema severities. As Figure 5c demonstrates, the first two raters (blue and green crosses) evenly included mild and severe erythema with the mean inter-rater *EI_BRG_* misestimate of 0.08. On the other hand, the third rater (black crosses) focused only on the severe erythema patches, resulting in the mean inter-rater *EI_BRG_* misestimate of 0.53, which represents an immense, almost six times error increase. Of course, such extreme inter-rater variability can be expected only in severe cases, where the erythema distribution is patchy [32]. On the other hand, *EI_BRG_* misestimates were merely around 0.01 (error of up to 1%) on the native or mildly erythematous skin (Table 5). In our previous report [19], readers can find a further discussion on the clinical relevance and limitations of the proposed dermatoscopic system in dogs.

Altogether, most of the misestimates in EI among raters were below 0.14, representing an error of up to 14% (canine *EI_BRG_* generally ranges from 1 to 2). In the previous study [29], we showed that different smartphone-based dermatoscopic systems had absolute disagreement in *EI_BRG_* for around 3%, significantly less than our veterinary raters. In the worst-case scenario, we could expect combined errors of up to 17%. Still, these errors were mostly not big enough to be detected by the human eye (Figure 6). Assuming that two other CIELAB dimensions (L*, b*) do not change, misestimates in a* were generally below 3.0, which is the lower range of JND, the color detection threshold for the human eye.

## 5. Conclusions

Our study showed that smartphone-based dermatoscopy is a convenient and reliable way to calculate EI and, by that, evaluate the severity of skin erythema in dogs with AD. We demonstrated a high correlation between the optical (*EI_BRG_*) and the dermatologists’ visual erythema evaluations with the average Spearman coefficient (*r_s_*) of 0.75. However, the proposed dermatoscopic approach should be applied to non-pigmented skin only since melanin can influence *EI_BRG_* level.

The tested manual ISMs exhibited high intra- and inter-rater reliabilities. Despite the method with selecting individual pixels (PT) achieving slightly better performance, we recommend selecting two small skin areas (SQ2 method), due to its speed. As with any ISM, there could be a significant inter-rater erythema misestimation on severely erythematous skin. In these cases, better outlined or automated ISMs should be tested to improve the erythema assessment.

## Figures and Tables

**Table 1:** Mean and standard deviation (SD) of the Spearman’s correlation coefficients (*r_s_*) between visual and optical erythema evaluation, relying on different erythema indices (EI: RG–*EI_RG_*_,_ GR–*EI_GR_*, BRG–*EI_BRG_*, BG–*EI_BG_*, and a*) and image-sampling methods (ISM: PT–60 pixels, SQ2–two small squares, SQ1–one big square).

ISM\EI	RG	GR	BRG	BG	a*	Mean
**PT**	0.73 ± 0.04	−0.73 ± 0.04	0.75 ± 0.05	0.66 ± 0.05	0.70 ± 0.03	0.71 ± 0.05
**SQ2**	0.72 ± 0.05	−0.72 ± 0.05	0.75 ± 0.05	0.67 ± 0.05	0.70 ± 0.05	0.71 ± 0.05
**SQ1**	0.70 ± 0.05	−0.70 ± 0.05	0.73 ± 0.05	0.61 ± 0.06	0.69 ± 0.04	0.68 ± 0.06
**Mean**	0.71 ± 0.05	−0.72 ± 0.05	0.74 ± 0.05	0.65 ± 0.06	0.70 ± 0.04	

**Table 2:** Mean and SD of the residuals (absolute values, in %) of linear regression models between visual and optical erythema evaluation, relying on different erythema indices (EI: RG–*EI_RG_*_,_ GR–*EI_GR_*, BRG–*EI_BRG_*, BG–*EI_BG_*, and a*) and image-sampling methods (ISM: PT–60 pixels, SQ2–two small squares, SQ1–one big square).

ISM\EI	RG	GR	BRG	BG	a*	Mean
**PT**	11.7 ± 0.7	11.7 ± 0.6	11.6 ± 0.5	12.7 ± 0.6	12.1 ± 0.3	12.0 ± 0.7
**SQ2**	11.9 ± 1.0	11.9 ± 0.8	11.6 ± 0.8	12.6 ± 0.8	12.3 ± 0.5	12.1 ± 0.8
**SQ1**	12.2 ± 0.7	12.3 ± 0.6	12.0 ± 0.5	12.9 ± 0.9	12.4 ± 0.3	12.4 ± 0.7
**Mean**	11.9 ± 0.8	12.0 ± 0.6	11.7 ± 0.6	12.7 ± 0.7	12.3 ± 0.4	

**Table 3:** Mean and SD of the intra- and inter-rater reliability coefficients (Spearman’s *r_s_*, intra-class correlation (ICC), and *α_k_*). Absolute agreement (*Δ*) is listed for two EIs: *EI_BRG_* and a* of the CIELAB color space.

Rater	Spearman	ICC (2, k)	*αk*	*ΔEI_BRG_*	Δa*
Intra-	0.97 ± 0.03	0.99 ± 0.01	0.98 ± 0.02	0.02 ± 0.02	0.6 ± 0.6
Inter-	0.94 ± 0.03	0.95 ± 0.04	0.90 ± 0.07	0.05 ± 0.09	1.3 ± 1.7

**Table 4:** Mean and SD of Krippendorff’s alpha (*α_k_*) when studying intra- and inter-rater reliability of individual ISMs (PT-60 pixels, SQ2-two small squares, SQ1-one big square).

Rater	PT	SQ2	SQ1
Intra-	0.99 ± 0.01	0.98 ± 0.01	0.97 ± 0.02
Inter-	0.88 ± 0.08	0.88 ± 0.07	0.93 ± 0.04

**Table 5:** Medians (with 2.5 and 97.5 percentiles in squared brackets) of *EI_BRG_* misestimates among raters for different erythema severity categories according to the Canine Atopic Dermatitis Extent and Severity Index, fourth iteration (CADESI4) scale.

Rater	0 (None)	1 (Mild)	2 (Moderate)	3 (Severe)
Intra-	0.01 [0.00–0.07]	0.01 [0.00–0.06]	0.02 [0.00–0.06]	0.03 [0.00–0.14]
Inter-	0.01 [0.00–0.33]	0.01 [0.00–0.22]	0.05 [0.00–0.22]	0.05 [0.00–0.60]

## Data Availability

Not applicable.
